# Transactivation of human *osteoprotegerin* promoter by GATA-3

**DOI:** 10.1038/srep12479

**Published:** 2015-07-28

**Authors:** Shyan-Yuan Kao, Konstantina M. Stankovic

**Affiliations:** 1 Eaton Peabody Laboratories and Department of Otolaryngology, Massachusetts Eye and Ear Infirmary; 2 Department of Otology and Laryngology, and Program in Speech and Hearing Bioscience and Technology, Harvard Medical School, Boston, Massachusetts, USA

## Abstract

Osteoprotegerin (OPG) is a key regulator of bone remodeling. Mutations in OPG are involved in a variety of human diseases. We have shown that cochlear spiral ganglion cells secrete OPG at high levels and lack of OPG causes sensorineural hearing loss in addition to the previously described conductive hearing loss. In order to study the regulation of OPG expression, we conducted a database search on regulatory elements in the promoter region of the *OPG* gene, and identified two potential GATA-3 binding sites. Using luciferase assays and site directed mutagenesis, we demonstrate that these two elements are GATA-3 responsive and support GATA-3 transactivation in human HEK and HeLa cells. The expression of wild type GATA-3 activated OPG mRNA and protein expression, while the expression of a dominant negative mutant of GATA-3 or a *GATA-3* shRNA construct reduced *OPG* mRNA and protein levels. *GATA-3* deficient cells generated by expressing a *GATA-3* shRNA construct were sensitive to apoptosis induced by etoposide and TNF-α. This apoptotic effect could be partly prevented by the co-treatment with exogenous OPG. Our results suggest new approaches to rescue diseases due to GATA-3 deficiency – such as in **h**ypoparathyroidism, sensorineural **d**eafness, and **r**enal (HDR) syndrome – by OPG therapy.

Osteoprotegerin (OPG) is a member of the tumor necrosis factor (TNF) receptor superfamily and a key regulator of bone metabolism. OPG acts as a soluble, neutralizing antagonist that competes with receptor activator of nuclear factor-κB (RANK) on preosteoclasts and osteoclasts for RANK ligand (RANKL) produced by osteoblasts. The discovery of the RANKL/RANK/OPG signaling and its role in the regulation of bone resorption, osteoclast formation, and skeletal remodeling represents a major advance in bone biology^1^. Using transgenic mice overexpressing OPG and rats injected with OPG, OPG has been shown to inhibit osteoclastogenesis *in vivo*^2^,^3^. OPG null mice displayed decreased total bone density and early-onset, severe osteoporosis accompanied with vascular calcification^4^,^5^, indicating that OPG is a negative regulator of osteoclastogenesis.

Besides RANKL, OPG is known to interact with another TNF family member, the TNF-related apoptosis inducing ligand (TRAIL). TRAIL can bind to its transmembrane receptors (death receptors: DR4 and DR5) to induce apoptosis in cancer cells^6^,^7^,^8^. OPG secreted by tumor cells or bone marrow stromal cells can efficiently bind to TRAIL and block its pro-apoptotic activity in a variety of tumor cell lines^6^,^7^,^8^. In addition to promoting cancer cell survival, OPG can induce angiogenesis^9^. Elevated OPG serum levels have been detected in patients with advanced malignancies such as prostate cancer^10^, bladder carcinoma^11^, and squamous cell head and neck cancer^12^. These observations confirm the role of OPG in tumor development.

We have shown that OPG null mice develop sensorineural hearing loss due to degeneration of spiral ganglion neurons^13^, in addition to the previously reported conductive hearing loss due to resorption of the bony ossicles in the middle ear^14^. Given the important roles of OPG in hearing, bone physiology, and tumor biology, we studied regulation of OPG expression by focusing on the *OPG* promoter. Others have demonstrated that the two Hox binding sites on human *OPG* promoter are responsible for bone morphogenesis protein (BMP) induced transcription of *OPG* gene and increased OPG secretion, thus inhibiting osteoclastogenesis^15^. Transforming growth factor-β (TGF-β) is known to activate human *OPG* promoter through the osteocalcin-specific element 2 (OSE2) and Smad binding element (SBE) sites, and this activation by TGF-β may be mediated by the interaction between Cbfa1 and Smad proteins^16^. Overexpression of OPG in human colorectal cancer cells is regulated by β-catenin and Tcf-4 bound to the human *OPG* promoter, thus mediating resistance to TRAIL-induced apoptosis in colon cancer^17^. We have explored OPG regulation by GATA-3, a member of the GATA family of transcription factors^18^ which bind to a GATA consensus motif (A/TGATAA/G)^19^.

We have focused on GATA-3 because mutations or deletions (haploinsufficiency) in the human *GATA3* gene cause sensorineural hearing loss as a part of the syndrome of hypoparathyroidism, sensorineural deafness, and renal disease (HDR syndrome)^20^,^21^,^22^,^23^. Moreover, manipulation of GATA-3 expression results in similar bone phenotype as seen when manipulating OPG expression. Specifically, inhibition of GATA-3 expression, by introducing *GATA3* small interfering RNA (siRNA) into osteoblasts, decreased GATA-3 levels and its downstream target bcl-x(L), thus causing apoptosis in osteoblasts^24^. Conversely, overexpression of GATA-3, in a mouse model of rheumatoid arthritis, protected against severe joint inflammation and bone erosion^25^.

GATA3 is highly conserved across vertebrate species. For example, the human and mouse GATA-3 proteins share a 96% amino acid homology^26^. GATA-3 is expressed in a variety of tissues in both human and mouse^27^, where it is involved in neuronal cell development and differentiation^28^, adipocyte differentiation^29^, T cell development^30^, skin cell differentiation^31^, mammary gland morphogenesis and luminal cell differentiation^32^, and lens development^33^. In addition, aberrant expression of GATA-3 is involved in a variety of malignancies in human and mouse models such as thymic lymphoma^34^, pancreatic cancer^35^, breast cancer^36^, and bladder cancer^37^.

We discovered several GATA-3 transcription factor binding sites on the promoter region of human *OPG* gene. The identification of OPG as a potential GATA-3 downstream target suggests that OPG and GATA-3 may share similar signaling pathways. This knowledge motivates future studies to determine whether exogenous OPG can partially rescue diseases due to GATA3 deficiency such as HDR syndrome, and whether the increase of OPG expression by manipulation of GATA3 can prevent diseases such as osteoporosis.

## Material and Methods

### Antibodies and reagents

The rabbit anti-GATA-3 polyclonal antibody (#5852), rabbit anti-cleaved caspase 3 (#9579), and the rabbit anti-β-actin polyclonal antibody (#4970) were purchased from Cell Signaling Technology (USA), the mouse anti-OPG antibody (ALX-804-532-C100) was from Enzo Life Sciences, DNA mutagenesis was performed using the QuikChange™ II Site-Directed Mutagenesis Kit (#200521, Agilent, USA) and the luciferase assay was performed using the Dual-Luciferase® Reporter Assay System (#E1910, Promega, USA). The DeadEnd™ Fluorometric TUNEL System was purchased from Promega (#G3250). The recombinant human osteoprotegerin was from R&D Systems (#185-OS-025/CF, USA). The apoptosis inducing agents, tumor necrosis factor alpha (TNF-α) (#8902) and etoposide (#341205), were from Cell Signaling and EMD Millipore (USA), respectively.

### Plasmids

**Figure 1:**
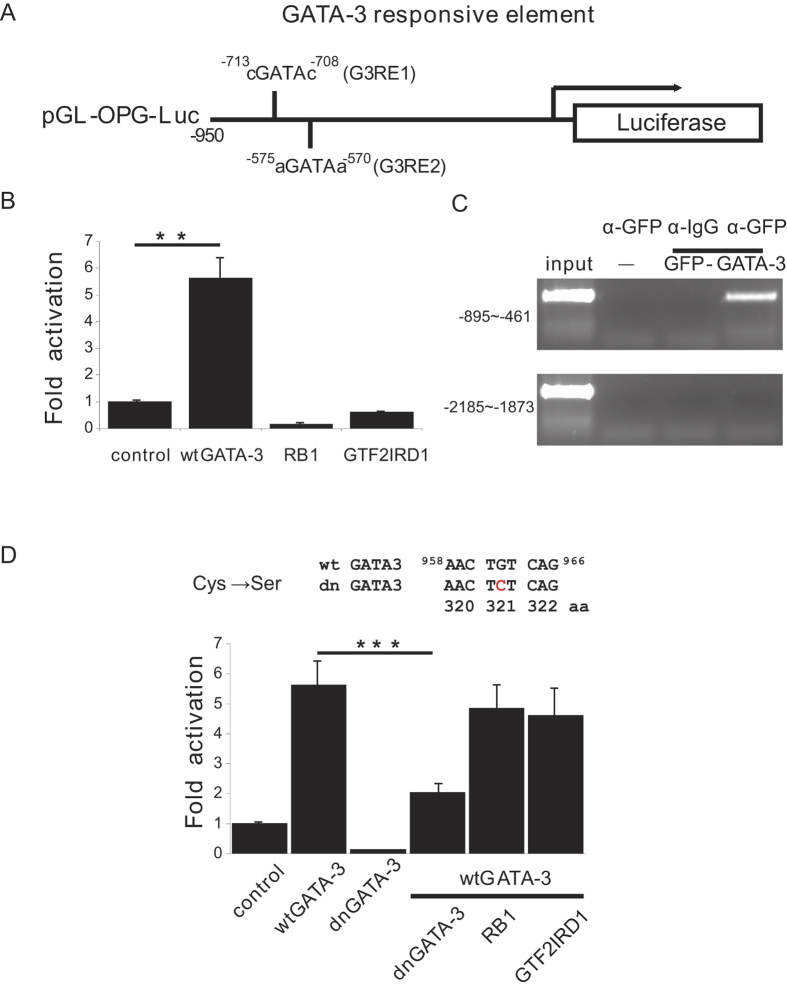
Transactivation of the *OPG* promoter by GATA-3. (**A**) A schematic diagram of two potential GATA-3 responsive elements on the human *OPG* promoter. The minimal *OPG* promoter was cloned from a HeLa genomic DNA library by PCR and cloned into the pGL-Luc vector. (**B**) Transactivation of the minimal *OPG* promoter by GATA-3 but neither RB-1 nor GTF2IRD1 using the dual luciferase assay. (**C**) Chromatin immunoprecipitation assay showing the binding of GATA-3 on the *OPG* promoter. The GFP-GATA-3 plasmid was transfected into HeLa cells and immunoprecipitated using an anti-GFP antibody. The PCR reaction showed that GFP-GATA-3 interacted with the chromatin containing the two potential GATA-3 responsive elements on the *OPG* promoter (from −895 to −461) but not the fragment outside the potential GATA-3 responsive element (from −2185 to −1873). (**D**) A dominant negative GATA-3 mutant lost its transactivation activity on the *OPG* promoter construct and could block the transactivation activity of the wild type GATA-3. **p < 0.01, ***p < 0.001, n = 5.

**Figure 2:**
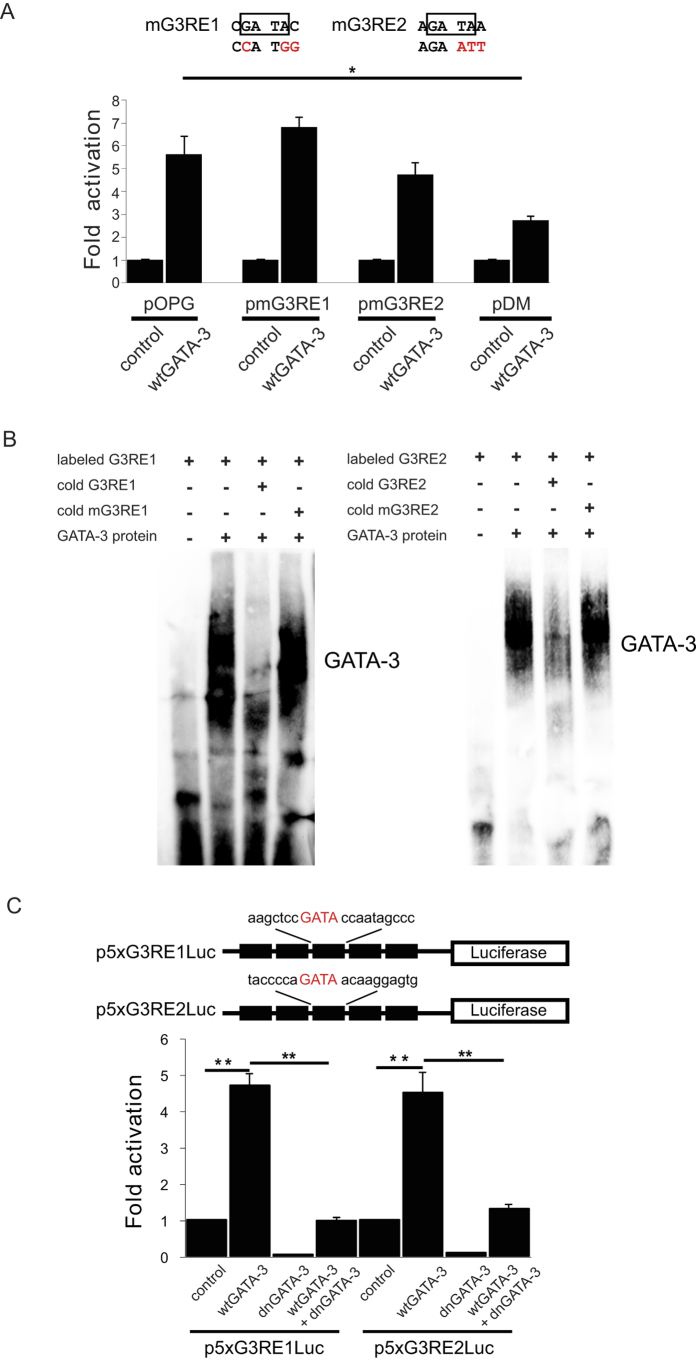
Identification of GATA-3 responsive elements on *OPG* promoter. (**A**) Luciferase results showed that mutations in either GATA-3 responsive element on the *OPG* promoter still supported GATA-3 transactivation while mutations in both elements blocked GATA-3 transactivation in HeLa cells after 24 hours of transfection. *p < 0.05, n = 5. (**B**) The gel shift assay showed that recombined GATA-3 could bind to the labeled G3RE1 and G3RE2 probes. This interaction could be blocked by a mutant G3RE1 or G3RE2 probe or excess non-labeled G3RE1 or G3RE2 probe. (**C**) The multiple minimal G3RE1 and G3RE2 could support GATA-3 transactivation. ** p < 0.01, n = 4.

The green fluorescent protein (GFP) tagged human GATA-3 plasmid, pcDNA-GFP-GATA-3, was a gift from Dr. Kwang-Soo Kim (McLean Hospital, Harvard Medical School). The dominant negative GATA-3 mutant^38^ was cloned from the wild type GATA-3 using the QuikChange™ Site-Directed Mutagenesis Kit to replace the cysteine residue at location 321 into serine. The internal control renilla plasmid, pRL-SV40, was purchased from Promega. The *OPG* promoter construct, pGL-OPG-Luc (pOPG), was amplified from HeLa total genomic DNA (Promega) by PCR and cloned into the pGL3 luciferase reporter vector, pGL3-basic (#E1751, Promega). The cloned *OPG* promoter sequence from HeLa cells was sequenced and no mutations were identified compared to the published *OPG* promoter sequence (Genebank accession number AY577781). The mutations on the *OPG* promoter (pmG3RE1, pmG3RE2, and pDM plasmids) were introduced using the QuikChange™ Site-Directed Mutagenesis Kit (Stratagene). The p5xG3RE1Luc and p5xG3RE2Luc were cloned using synthetic oligos. The sequences were shown in Figs 1 and 2 (GenBank accession number AY466112). The GATA3 specific shRNA (sc-29331-SH) and control shRNA plasmid-A (sc-108060) were from Santa Cruz Biotechnology. pSG5L-HA-RB (#10720, Addgene) and pEBB-GFP-hBEN (#22157, Addgene) were used as controls for non-specific transcriptional regulators which did not bind to the GATA-3 binding site.

### Luciferase assay

The human embryonic kidney (HEK 293) cells were transfected with the *OPG* promoter reporter, pcDNA-GFP-GATA-3, or pcDNA-dnGATA-3 plasmids together with the pRL-SV40 (to control for transfection efficiency). Twenty-four hours after transfection, cells were lysed and luciferase and renilla activity were measured using a Wallac VICTOR Model 1420 multilabel counter (PerkinElmer). Luciferase activity was normalized to the renilla activity of the same plate. The fold induction was determined as the average of three wells per treatment normalized to the average signal of the reporter-alone wells, which was set as 1.

### Chromatin immunoprecipitation

The association between GATA-3 and the *OPG* promoter was assayed by chromatin immunoprecipitation (ChIP) using the ChIP assay protocol described before^39^ with some modifications. HeLa cells transfected with pGFP-GATA-3 or a control plasmid were harvested and the amount of DNA in the resulting cell lysates was quantified by measuring absorption at 260 nm; the DNA concentration was then normalized to 100 μg/μl. Supernatant (200 μg DNA) was diluted 10-fold in 2 ml ChIP dilution buffer (0.01% SDS, 1.1% Triton X-100, 1.2 mM EDTA, 16.7 mM Tris-HCl, pH 8.1, 167 mM NaCl, and protease inhibitors), and precleared twice with BSA-blocked Protein A/G Agarose (sc-2003, Santa Cruz) (2 × 100 μg, 2 × 30 min at 4 °C). The beads were centrifuged and the supernatant was divided into 4 × 500 μl aliquots for immunoprecipitation, input DNA, and the IgG control. Primary antibody was added and incubated at 4 °C overnight. A rabbit anti-GFP polyclonal antibody (A6455, Invitrogen) was used for immunoprecipitation of GATA-3, and ChromPure rabbit IgG (#011-000-003, Jackson ImmunoResearch) was used for the IgG control. Thirty μl of BSA-blocked Protein A/G Agarose was then added and incubated at 4 °C with rotation. The beads were centrifuged and washed once with a low salt immune complex buffer (#20–154, Millipore), twice with a high salt wash buffer, once with a LiCl wash buffer (#20–156, Millipore), and twice in TE buffer (10 mM Tris-HCl, 1 mM EDTA, pH 8.0). The washed agarose beads were eluted with 2 × 250 μl freshly prepared elution buffer (1% SDS, 0.1 mM NaHCO3). DNA crosslinking was reversed by adding 5 M NaCl and heating at 65 °C for 4 hrs. Protein was removed by incubation with 20 mg/ml proteinase K in 10 μg EDTA/40 mM Tris-HCl, pH 6.5 for 3 hr at 45 °C. De-crosslinked DNA was isolated by QIAquick PCR purification kit (#28104, Qiagen) and ethanol precipitation. The precipitated DNA was washed with 70% ethanol, air dried and dissolved in ddH2O for PCR. The amplification product by primers for GATA binding sites in the *OPG* promoter was from −895 to −461 of the *OPG* promoter, and the amplification product by control primers was from −2185 to −1873 of the *OPG* promoter.

### Real-time quantitative RT-PCR

HEK cells were transfected with wild type GATA-3, dominant negative GATA-3, empty pcDNA3.1 expressing plasmids, GATA-3 shRNA, or control shRNA plasmid-A.

After 48 h of transfection, total RNA was extracted using RNeasy Mini Kit (#74104, Qiagen) according to the manufacturer’s protocol. Total RNA was reverse transcribed with Taqman Reverse Transcription Reagents kit (N8080234, Life Technologies). Real-time quantitative RT-PCR (qRT-PCR) was performed using 6-FAM linked fluorescent probes and primers for human *OPG* (#4331182), and *GATA-3* (#4331182) designed and optimized by Applied Biosystems. The measurements were carried out on the Mx3005P machine (Stratagene) using 96 well plates. Fluorescence data were collected starting with a denaturation step at 95 °C for 10 minutes, followed by 45 cycles of 95 °C for 15 seconds and 60 °C for 1 minute. Gene expression levels were quantified relative to the 18 S rRNA gene, and analyzed using the comparative threshold cycle method. Previous studies have shown that GATA-3 does not activate 18 S gene^40^.

### Gel shift assay

The gel shift assay was performed using the LightShift^TM^ EMSA Optimization & Control Kit (#20148, Thermo Scientific) according to the manufacturer’s manual. The purified human GATA-3 protein was from OriGene. The 6% DNA retardation gels, biotin labeled G3RE1 probe (5'-TAGGAAGCTCCGATACCAATAGCCCT-3'), unlabeled G3RE1 probe, unlabeled mutant G3RE1 probe (5'-TAGGAAGCTCCCATGGCAATAGCCCT-3'), wild type G3RE2 probe (5'-TAACTACCCCAGATAAGAAGGAGTGA-3'), unlabeled wild type G3RE2 probe, and unlabeled mutant G3RE2 probe (5'-TAACTACCCCAGAGTTGAAGGAGTGA-3') were synthesized by Invitrogen.

### Western blot analysis

Cells were lysed in RIPA-DOC buffer (50 mM Tris buffer (pH 7.2), 150 mM NaCl, 1% Triton-X100, 1% deoxycholate and 0.1% SDS) with protease inhibitors (cOmplete, # 04693132001, Roche Molecular Biochemicals). Equal amount of protein extract was loaded per lane, resolved by 4–20% SDS–PAGE, and electro-transferred onto a PVDF membrane (IPVH00010, Immobilon-P, Millipore). Detection of proteins by antibodies was performed as described previously^41^. Immunoreactivity was detected with the enhanced chemiluminescence detection kit (#32106, ECL, Pierce). Protein bands were quantitated using the ChemiDoc™ XRS+ System. Actin was used as the internal control because previous studies have shown that GATA-3 does not activate actin expression^17^,^30^.

### Enzyme-linked immunosorbent assay (ELISA) for OPG in cell lysate and culture medium

Detection of OPG secreted from culture cells was performed using the Human OPG ELISA kit (ELH-OPG, RayBiotech) according to the instruction manual. For OPG in cell lysate, cells were first suspended in cell lysis buffer (50 mM Tris-HCl (pH 8.0), 150 nm NaCl, 2 mM EDTA, and 1% NP40) followed by sonication. For OPG in culture medium, cells were cultured overnight and the culture medium was collected for ELISA. The OPG concentration in cell lysate was presented as the amount of OPG protein in total cell lysate. The OPG concentration in culture medium was presented as the level of OPG concentration in the culture medium per 1000 μg of cell lysate.

### Detection of apoptosis

Cells were pre-treated with OPG (10 μg/mL) for 2 h followed by co-treatment with TNF-α (2 μg/mL) or etoposide (10 μM) overnight. For detection of cleaved caspase 3, cells were harvested for western blot analysis as described above, and the rabbit anti-caspase 3 antibody (Cell Signaling) was used. For terminal deoxynucleotidyl transferase dUTP nick end labeling (TUNEL) assay, cells were fixed with 4% paraformaldehyde after treatment and the assay was performed according to the operation manual of the DeadEnd™ Fluorometric TUNEL System (G3250, Promega).

### Statistical analysis

Data were presented as mean ± standard deviation and analyzed using Excel (Microsoft). Data within a group were compared using the paired t test and data between groups were compared using the analysis of variance. Differences were considered significant if p < 0.05.

## Results

### Transactivation of the *OPG* promoter by GATA-3

To study the regulation of the human OPG expression, the human *OP*G promoter was analyzed using several on-line promoter and transcription factor analysis tools including Tfsitescan (http://www.ifti.org/Tfsitescan/) and PROMO (http://alggen.lsi.upc.es/cgi-bin/promo_v3/promo/promoinit.cgi?dirDB=TF_8.3. The common feature of these analyses was identification of two potential GATA-3 binding sites on human *OPG* promoter at −713 to −708 (which we named GATA-3 responsive 1, G3RE1) and −575 to −570 (which we named G3RE2) (GenBank accession number: AY466112) (Fig. 1A). To test whether these two putative GATA-3 responsive elements on the *OPG* promoter could support GATA-3 transactivation, we cloned the promoter sequence from the HeLa total genomic DNA into the luciferase reporter vector pGL-basic. Luciferase assays were performed to determine the ability of GATA-3 to activate the *OPG* promoter. The GATA-3 transactivated the *OPG* promoter 5.61 ± 0.79 fold (n = 5, p = 0.002), while the control transcriptional factors, retinoblastoma 1 (RB1) and general transcription factor II-I repeat domain-containing protein 1 (GTF2IRD1), which do not bind to the GATA-3 binding site, did not activate the *OPG* promoter (Fig. 1B).

To further validate the transactivation ability of GATA-3 on *OPG* promoter, a ChiP assay was performed. The chromatin fragment of the *OPG* promoter region from −895 ~ −461, containing both the G3RE1 and G3RE2 sites, could be immunoprecipitated by the GFP antibody after transfection with the pGFP-GATA-3 plasmid (Fig. 1C, top). In the control, the chromatin fragment of the *OPG* promoter without GATA-3 binding sites (from −2185 to −1873) could not be immunoprecipitated when the pGFP-GATA-3 plasmid was transfected (Fig. 1C, bottom). These results suggest that GATA-3 could transactivate the human OPG promoter through the two GATA-3 binding sites.

To further test the specificity of the GATA-3 binding sites, a GATA-3 dominant negative mutant was cloned using the PCR mutagenesis method. The GATA-3 dominant negative mutant (dnGATA-3) was discovered in an HDR patient, which had a cysteine to serine mutation at the position of amino acid 321 (Fig. 1D, top)^38^. The dnGATA-3 lacked transactivation activity due to the loss of DNA binding activity and disrupted the transactivation activity of the wild type GATA-3 (wtGATA-3). The luciferase results showed that the dnGATA-3 failed to transactivate the *OPG* promoter and blocked the transactivation activity of wtGATA-3 with the *OPG* promoter when wtGATA-3 and dnGATA-3 were co-transfected with the *OPG* promoter reporter (n = 5, p = 0.0007). The control RB1 and GTF2IRD1 proteins had little effect on the transactivation activity of the wtGATA-3 (Fig. 1D, bottom).

### Characterization of minimal GATA-3 responsive elements on the *OPG* promoter

To determine whether the two putative GATA-3 binding sites on the *OPG* promoter were true GATA-3 binding sites, several mutations were introduced into the distal site, proximal site, or both sites to make the pmG3RE1, pmG3RE2, and pDM plasmids, respectively (Fig. 2A, top). The luciferase results showed that mutations on either binding site did not significantly disrupt the transactivation by the wtGATA-3 (p = 0.83 for pmG3RE1 and p = 0.28 for pmG3RE2 compared to pOPG; n = 5) (Fig. 2A). However, mutations at both sites, pDM, significantly decreased the transactivation by the wtGATA3 (p = 0.03). These results suggest that these two GATA-3 binding sites have redundant GATA-3 binding function so that loss of both precludes binding.

To further demonstrate that G3RE1 or G3RE2 alone could support GATA-3 transactivation, the gel shift assay was performed. When incubating the biotin-labeled probes containing either G3RE1 or G3RE2 with the HeLa nuclear extract, a specific band was observed (Fig. 2B, lane 2 and lane 6). This band disappeared by adding excess un-labeled G3RE1 or G3RE2 probes (Fig. 2B, lane 3 and lane 7) but not by adding excess cold (non-labeled) mutant G3RE1 or G3RE2 probes (Fig. 2B, lane 4 and lane 8). These results suggest that the G3RE1 and G3RE2 probe could bind GATA3 protein in HeLa nuclear extract. In order to further support that these minimal elements could support GATA-3 activation, we constructed two reporter plasmids with minimal 5-fold GATA-3 responsive element, p5xG3RE1Luc and p5xG3RE2Luc (Fig. 2C, top). A luciferase assay showed that these two constructs with minimal GATA-3 responsive elements from the *OPG* promoter supported GATA-3 transactivation (Fig. 2C, bottom) by increasing activity 4.70 ± 0.34 fold for G3RE1 (p = 0.0016, n = 4) and 4.50 ± 0.61 fold for G3RE2 (p = 0.01, n = 4). The activation of the two constructs with minimal GATA-3 responsive elements by wtGATA-3 could be disrupted by the co-transfection of the dominant negative GATA-3 mutant (p = 0.001 and 0.01, respectively, n = 4), further indicating that GATA-3 transactivates the *OPG* promoter through these two elements.

### Regulation of the expression of the *OPG* gene and protein by GATA-3

**Figure 3:**
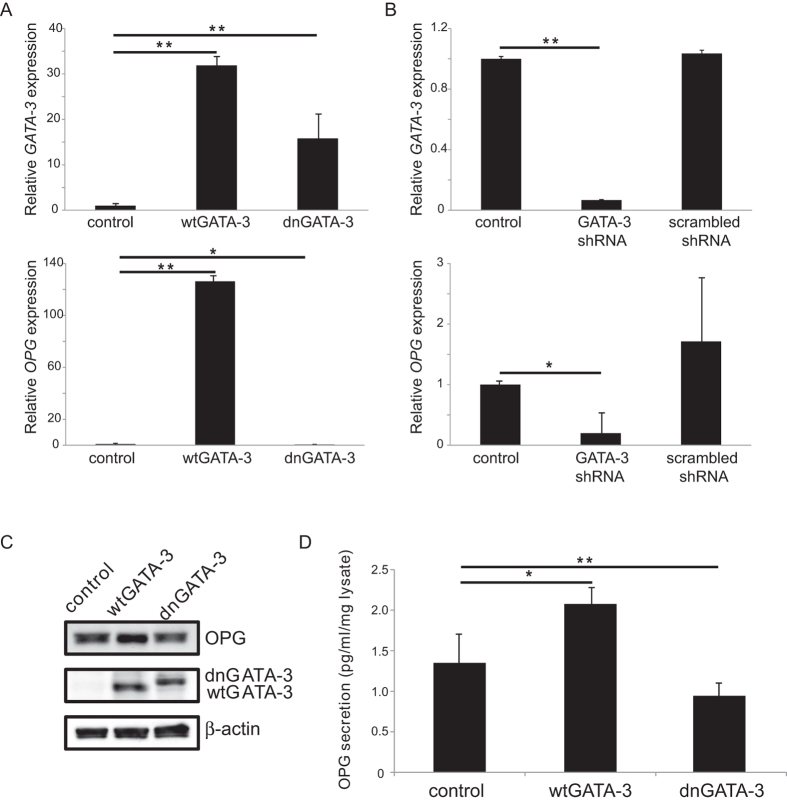
Regulation of endogenous *OPG* mRNA and protein by GATA-3. (**A**) wtGATA-3 and dnGATA-3 expression plasmids were transfected into HEK cells and the expression of *GATA-*3 and *OPG* mRNA was measured using qRT-PCR. (**B**) *GATA-3* and scrambled shRNA constructs were transfected into HEK cells and *GATA-3* and *OPG* mRNA expression after 48 h was measured using qRT-PCR. (**C**) wtGATA-3 and dnGATA-3 expression plasmids were transfected into HEK cells and the expression of GATA-3 and OPG proteins was detected using western blot analysis.(**D**) OPG secretion from HEK cells transfected with either the wtGATA-3 or dnGATA-3 plasmid for 48 h were measured in cell-conditioned culture medium using ELISA. *p < 0.05, **p < 0.01, n = 3 for qRT-PCR and western blot and n = 4 for ELISA. Full-length blots are presented in supplementary Figure 1.

The effect of GATA-3 on the expression of the *OPG* gene and protein *in vivo* was investigated using the qRT-PCR (Fig. 3A,B) and western blot analysis (Fig. 3C). HEK cells were transfected with the wtGATA-3 plasmid, the dnGATA-3 plasmid, GATA-3 shRNA, scrambled shRNA, or an empty vector. Forty-eight hours after transfection, cells were harvested, and total RNA (n = 3 different cultures) or total protein (n = 3 different cultures) was isolated. The qRT-PCR results showed that overexpression of wtGATA-3 increased the expression of the endogenous *OPG* mRNA 126.24 ± 4.84 fold (p = 0.0001), while the overexpression of the dnGATA-3 reduced the expression of *OPG* mRNA by 0.5297 ± 0.18 fold (p = 0.022) relative to controls (set at 1) transfected with the empty vector (Fig. 3A). The transfection of *GATA-3* shRNA reduced the expression of endogenous *GATA-3* mRNA (0.067 ± 0.004, p = 0.0034) and *OPG* (0.199 ± 0.35, p = 0.042). The transfection of scrambled shRNA had no statistically significant effect on the expression of endogenous *GATA-3* mRNA (1.036 ± 0.021, p = 0.15) or *OPG* mRNA (1.7144 ± 1.05, p = 0.32) (Fig. 3B). At the protein level, the expression of endogenous OPG in cultured HeLa cells increased 1.83 ± 0.32 fold (p = 0.003) after overexpression of the wild type GATA-3, while the expression of OPG was reduced by 0.85 ± 0.10 fold (p = 0.033) following the overexpression of the dominant negative GATA-3 compared to controls (set at 1) (Fig. 3C). These results indicate that, in addition to activating the minimal *OPG* promoter construct, GATA-3 also activated the expression of the endogenous *OPG* mRNA and protein.

To further quantify the level of OPG expression in cultured cells and their secretions, ELISA was applied to cell lysates and conditioned culture medium after transfecting cells with the wtGATA-3, dnGATA-3, or a GATA-3 specific shRNA (Fig. 3D). Because the level of OPG secretion from HeLa cells was higher than the highest OPG standard in the ELISA kit, it was difficult to compare the differences after transfection with either wtGATA-3 or dnGATA-3 expressing plasmids. Therefore, we used HEK-293 cells whose baseline secretion of OPG was 1.42 ± 0.31 pg/ml per 1 mg cell lysate (n = 4 different cultures). After transfection with the wtGATA-3 expressing plasmid, the level of OPG expression increased to 2.08 ± 0.26 pg/ml per 1 ml cell lysate (p = 0.02). In contrast, the transfection of dnGATA-3 expressing plasmid reduced the level of OPG expression to 0.94 ± 0.21 pg/ml per 1 ml cell lysate (p = 0.01). These results further confirm that *OPG* is a target gene for GATA-3.

### Regulation of cell death by GATA-3 and OPG

**Figure 4:**
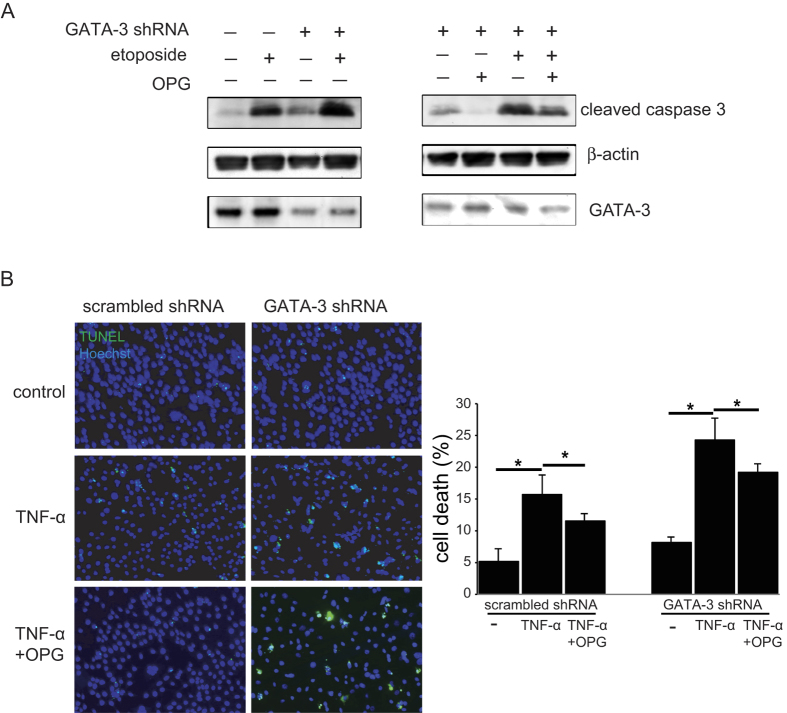
Regulation of apoptosis by GATA-3 and OPG. (**A**) HEK cells expressing either a scrambled shRNA or a *GATA-3* shRNA expressing plasmid were treated with etoposide (10 μM), and apoptosis was assayed by Western blot (left). HEK cells expressing a *GATA-3* shRNA expressing plasmid were treated with purified OPG protein for 2 h before treatment with etoposide, and apoptosis was assayed by Western blot (right). Full-length blots are presented in supplementary Figure 1. (**B**) HEK cells expressing either a control plasmid or a *GATA-3* shRNA expressing plasmid were treated with TNF-α (2 μg/mL) with or without pre-treatment with OPG for 2 h. Subsequent cell death was measured using TUNEL assays (left), and quantified with bar graphs (right). *p < 0.05, n = 3.

To study the effect of activation of OPG by GATA-3, a GATA-3 deficient HEK cell line was established by introducing a GATA-3 specific shRNA. The expression of the shRNA construct reduced the expression of GATA-3 protein in HEK cells, which resulted in the reduction of OPG protein expression (Fig. 4A, bottom left). Compared with the control cells which expressed the control scrambled shRNA plasmid (Fig. 4A, left), the GATA-3 shRNA expressing cells exhibited a higher level of cell death, as shown in the increased expression of cleaved caspase 3 protein in the western blot analysis (Fig. 4A, left, column 3 versus 1). When treated with etoposide, a topoisomerase inhibitor that induces apoptotic cell death, the level of cell death in the GATA-3 shRNA expressing cells was even more evident compared with that in the control cells (Fig. 4A, left, column 4 versus 2). These results indicate that GATA-3 deficient cells were more prone to apoptotic cell death. This increased cell death in GATA-3 shRNA expressing cells could be suppressed by the addition of exogenous recombinant OPG protein (Fig. 4A, right). The cell death induced by etoposide in GATA-3 deficient cells could also be suppressed by OPG (Fig. 4A, right).

To further confirm that cell death mediated by GATA-3 deficiency could be suppressed by the addition of the exogenous recombinant OPG proteins, a different apoptosis inducing agent and a different apoptosis detection method were used. The TUNEL assay showed that without treatment, the level of cell death in GATA-3 shRNA expressing cells (8.17 ± 0.82%) was higher than that in the control cells (5.19 ± 1.85%) (Fig. 4B, p = 0.044). The difference in the levels of cell death was enhanced when cells were treated with TNF-α (Fig. 4B), raising the levels from from 16.38 ± 2.52% in the control cells to 24.30 ± 2.63 in the GATA-3 shRNA treated cells (p = 0.045) . The increased levels of cell death in GATA-3 deficient cells could be suppressed to 18.20 ± 1.2 (p = 0.04) by the addition of recombinant OPG protein (Fig. 4B), suggesting that OPG is a downstream molecule of GATA-3, and that OPG can partially rescue the function of GATA-3 deficiency.

## Discussion

Using a variety of molecular approaches, including site directed mutagenesis and cell transfection with mutant GATA-3 constructs, we have shown for the first time that GATA-3 transactivates the human *OPG* promoter. Although we have focused on cell lines in these mechanistic studies, our results have implications for a variety of physiologic and pathologic processes that involve OPG and GATA-3.

We are particularly interested in our findings’ implication for bone metabolism and sensorineural hearing loss. With regard to bone metabolism, our results provide insight into the observed similarity of bone phenotypes due to OPG deficiency and GATA-3 deficiency. Specifically, OPG null mice have severe osteoporosis due to increased osteoclastogenesis^4^,^5^ whereas GATA-3 inhibition in osteoblasts induces their apoptosis^24^. Conversely, exogenous OPG can successfully treat osteoporosis^42^ whereas GATA-3 overexpression protects against bone erosion in arthritis^24^. These observations, in light of our findings, suggest a mechanism for overlapping functions of GATA-3 and OPG.

Our results may also shed light on the unclear mechanism of sensorineural hearing loss due to GATA-3 deficiency in HDR syndrome. Sensorineural hearing loss in HDR syndrome occurs at birth and is bilateral^21^. Histopathology of the human inner ear has not been described in HDR syndrome. However, a critical role of GATA-3 in the development of the mammalian inner ear and auditory neurons has been demonstrated in animal models^43^,^44^,^45^. GATA-3 is expressed in the murine optic placode as early as embryonic day 9 (E9)^43^, and it is expressed in both sensory and nonsensory cells, including cells in the developing cochlear duct and spiral ganglion^44^. Expression of GATA-3 in the organ of Corti and spiral ganglion persists throughout adulthood^44^. *Gata3* haploinsufficiency in mice causes progressive degeneration of hair cells, supporting cells, and spiral ganglion neurons in the cochlea, which is reflected in physiologic metrics of elevated auditory brainstem response thresholds and decreased distortion product otoacoustic emissions^44^,^46^. Loss of GATA-3 expression also causes limited morphogenesis and neurosensory development, altered inner ear innervation, and reduced projection of vestibulo-cochlear efferents in the murine cochlea^47^. GATA-3 controls prosensory domain specification and regulates survival of spiral ganglion neurons in the murine cochlea^48^. GATA-3 is considered an “intermediate regulator” of spiral ganglion neuron differentiation, and is critical for cochlear wiring^49^.

Despite the important role of the transcription factor GATA-3 in inner ear development and function, the downstream targets of GATA-3 in the inner ear or cochlear neurons have not been identified. Although several studies have attempted this identification^40^,^50^, the target genes of GATA-3 in the cochlea are not fully elucidated. In this study, we identified two GATA-3 responsive elements on the *OPG* promoter, and demonstrated regulation of OPG expression by GATA-3. These results may provide insight into the function of GATA-3 and OPG in the cochlea. OPG is present at exceptionally high levels in cochlear fluids and tissues compared to those in cerebrospinal fluid (CSF), serum and bone^51^ – the finding that was unexpected as previous studies had focused on the role of OPG in bone development. Mice lacking functional OPG develop progressive hearing loss^52^. We have shown that this hearing loss is due not only to abnormalities in the bone surrounding the inner ear but, even more importantly, to the degeneration of spiral ganglion neurons^13^. These neurons highly express and secrete OPG. We have also shown that OPG is important for the proliferation and survival of inner ear stem cells^13^. These OPG functions in the cochlea overlap with GATA-3 functions in the same tissues. These published results – combined with our current findings of transactivation of *OPG* promoter by GATA-3 and partial rescue of cell death mediated by reduced GATA-3 expression by OPG overexpression – strongly motivate future experiments to determine whether OPG can partially rescue hearing loss due to GATA-3 deficiency, as seen in HDR syndrome.

## Additional Information

**How to cite this article**: Kao, S.-Y. and Stankovic, K. M. Transactivation of human *osteoprotegerin* promoter by GATA-3. *Sci. Rep.* **5**, 12479; doi: 10.1038/srep12479 (2015).

## Supplementary Material

Supplementary Information
