# Supplementary material for: Transactivation of human osteoprotegerin promoter by GATA-3
Source: Sci Rep. 2015 Jul 28;5:12479. doi: 10.1038/srep12479 (PMC4516985; doi:10.1038/srep12479)
Supplement: Supplementary Information [file srep12479-s1.pdf]

Supplementary information

for

Transactivation of human *osteoprotegerin* promoter by GATA-3

[1]Shyan-Yuan Kao and [1,2]Konstantina M. Stankovic

[1]Eaton Peabody Laboratories and Department of Otolaryngology, Massachusetts Eye and Ear Infirmary, [2]Department of Otology and Laryngology, and Program in Speech and Hearing Bioscience and Technology, Harvard Medical School, Boston, Massachusetts, USA

.

Corresponding author:

Konstantina Stankovic

Massachusetts Eye and Ear Infirmary

243 Charles Street

Boston, MA, 02114-3096

Tel: 617 573 3972

Fax: 617 573 3939

Email: konstantina_stankovic@meei.harvard.edu

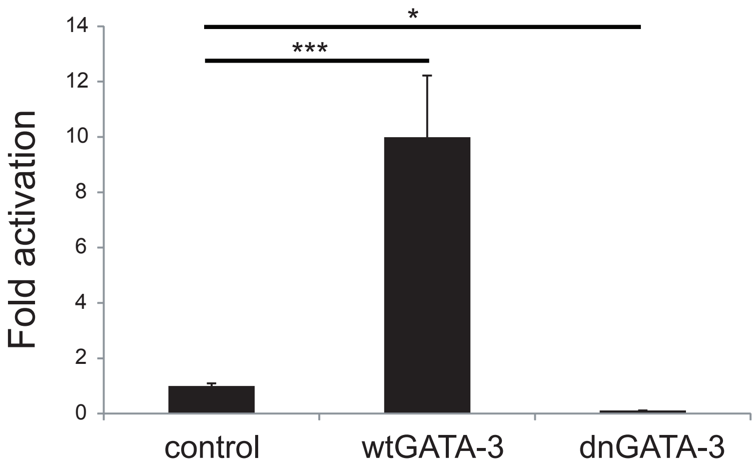

Supplementary Figure 1. Transactivation of OPG promoter by GATA-3 in HEK cells. Transactivation of OPG promoter by wtGATA-3 but not dnGATA-3 in HEK cells. *p<0.05, ***p<0.001, n=3.

Fig 3B

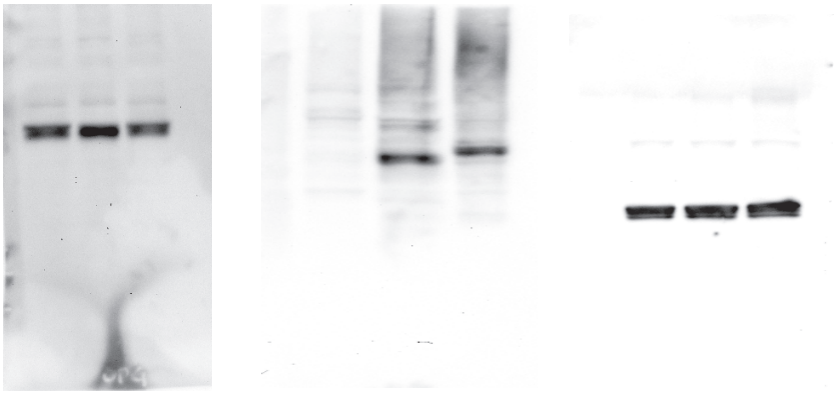

OPG GATA-3 β-actin

Fig 4A

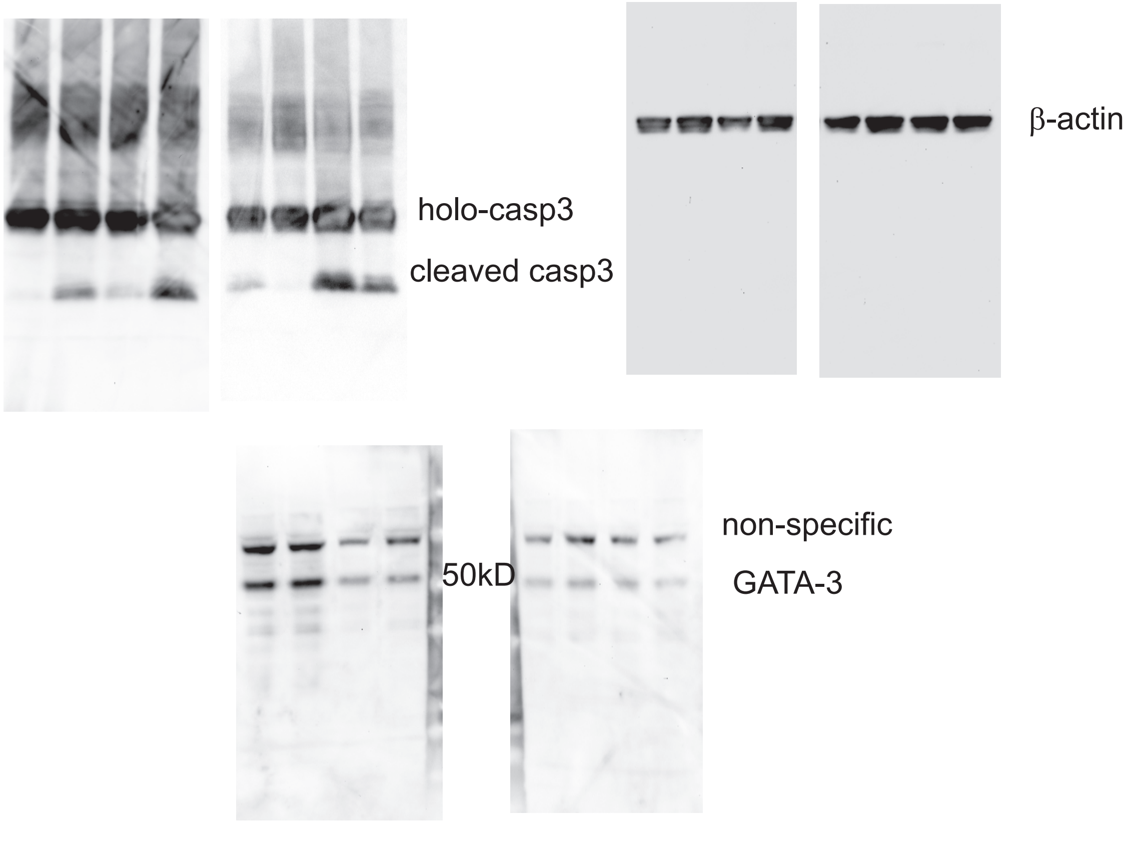

Supplementary Figure 2. Full blot and gel image
